# The efficacy and value of emergency medicine: a supportive literature review

**DOI:** 10.1186/1865-1380-4-44

**Published:** 2011-07-22

**Authors:** C James Holliman, Terrence M Mulligan, Robert E Suter, Peter Cameron, Lee Wallis, Philip D Anderson, Kathleen Clem

**Affiliations:** 1 The Center for Disaster and Humanitarian Assistance Medicine, Uniformed Services University of the Health Sciences, and George Washington University School of Medicine and Health Sciences, Bethesda, MD, USA; 2 The Department of Emergency Medicine, University of Maryland School of Medicine, Baltimore, MD, USA; 3 The Department of Military and Emergency Medicine, Uniformed Services University of the Health Sciences, Bethesda, MD, USA, and the Division of Emergency Medicine, University of Texas Southwestern, Dallas, TX, USA; 4 The Department of Emergency Medicine, the Alfred Hospital, Monash University, Melbourne, Australia; 5 The Division of Emergency Medicine, Stellenbosch University, Capetown, South Africa; 6 The Department of Emergency Medicine, Beth Israel Deaconess Medical Center and the Harvard Medical School, Boston, MA, USA; 7 The Department of Emergency Medicine, Loma Linda University, Loma Linda, CA, USA

## Abstract

**Study objectives:**

The goal of this study was to identify publications in the medical literature that support the efficacy or value of Emergency Medicine (EM) as a medical specialty and of clinical care delivered by trained emergency physicians. In this study we use the term "value" to refer both to the "efficacy of clinical care" in terms of achieving desired patient outcomes, as well as "efficiency" in terms of effective and/or cost-effective utilization of healthcare resources in delivering emergency care. A comprehensive listing of publications describing the efficacy or value of EM has not been previously published. It is anticipated that the accumulated reference list generated by this study will serve to help promote awareness of the value of EM as a medical specialty, and acceptance and development of the specialty of EM in countries where EM is new or not yet fully established.

**Methods:**

The January 1995 to October 2010 issues of selected journals, including the EM journals with the highest article impact factors, were reviewed to identify articles of studies or commentaries that evaluated efficacy, effectiveness, and/or value related to EM as a specialty or to clinical care delivered by EM practitioners. Articles were included if they found a positive or beneficial effect of EM or of EM physician-provided medical care. Additional articles that had been published prior to 1995 or in other non-EM journals already known to the authors were also included.

**Results:**

A total of 282 articles were identified, and each was categorized into one of the following topics: efficacy of EM for critical care and procedures (31 articles), efficacy of EM for efficiency or cost of care (30 articles), efficacy of EM for public health or preventive medicine (34 articles), efficacy of EM for radiology (11 articles), efficacy of EM for trauma or airway management (27 articles), efficacy of EM for using ultrasound (56 articles), efficacy of EM faculty (34 articles), efficacy of EM residencies (24 articles), and overviews and editorials of EM efficacy and value (35 articles).

**Conclusion:**

There is extensive medical literature that supports the efficacy and value for both EM as a medical specialty and for emergency patient care delivered by trained EM physicians.

## Introduction

### Background

Emergency Medicine (EM) is an officially recognized medical specialty in over 60 countries, with the rate of specialty recognition accelerating in recent years [1]. Recent epidemiologic and demographic public health data highlight the growing need for EM, trauma, and acute care development in all countries across the socioeconomic spectrum. According to the 2006 World Health Organization studies on the Global Burden of Disease [2], worldwide demographic and epidemiologic shifts now show non-communicable diseases to have become the single largest cause of morbidity and mortality worldwide. Stroke, cardiovascular disease, cancer, and trauma have, for the first time, surpassed communicable diseases and are listed as the major global causes of death and disability. EM care delivery systems are specifically focused on managing the acute consequences of non-communicable as well as communicable disease processes, and therefore represent an important public health tool for reducing the present and future global disease burden, similar to the effect that immunization programs and other public health initiatives have had on communicable disease in the past [3]. In countries where EM has been well established as a medical specialty for decades, the value of EM specialty training and an emergency care system staffed by EM specialists may seem self-evident to all stakeholders within the healthcare system; however, in countries where EM is still not yet well established or recognized as a medical specialty, this is often not the case. Opponents of EM specialty recognition in countries without EM have argued that there is no scientific evidence that an EM specialty-based emergency care system would provide measureable benefit. EM specialist physicians and other advocates for better emergency care in countries with established EM specialties have long understood and accepted the relationship between EM specialty recognition, EM specialty training and improved emergency care delivery. Therefore, there has not been a motivation or need in these countries to systematically prove the value and benefit of EM specialty-based emergency care systems as compared with non-EM specialty-based emergency care systems any more than other established medical specialties are compelled to justify their existence. However, in countries where EM is new or not yet recognized, there may be a lack of awareness of the benefits of EM specialty-based emergency care among healthcare policy and decision makers, so there is a need to systematically summarize the evidence supporting the benefit of EM, in order to facilitate the process of gaining official approval, adoption, or recognition of EM as a specialty.

In countries where EM has been well established and officially recognized, EM has such close and extensive interactions with the rest of the health care system and with other specialties that documenting the direct effects of EM alone is problematic. Performing "before and after" studies of the efficacy of newly introducing EM to many countries has also been challenging because of the poor quality and reliability of health outcome data in these countries prior to the introduction of EM. Despite these challenges, the authors found that an extensive body of medical literature has been published over the past several decades that supports the value of EM specialty-based emergency care delivery systems. The main aim of the project reported in this manuscript is to correct the misconception that there have been few publications to date that support the value of EM.

### Importance

The authors undertook the "Efficacy of EM Project" described in this manuscript to provide compiled reference articles that support EM efficacy and value. It is hoped that this review of some of the supportive literature and references will be useful in promoting the establishment, recognition, and continued development of EM in countries where the specialty is still forming, as well as in countries where it already exists.

### Goals

The goals of this study were to accumulate a reference list of articles from selected medical journals that supported the efficacy, effectiveness, an or value of EM as a medical specialty or of clinical care delivered by trained EM physicians. In this study we use the term "value" to refer both to the "efficacy of clinical care" in terms of achieving desired patient outcomes as well as "efficiency" in terms of effective and/or cost-effective utilization of healthcare resources in delivering emergency care.

## Methods

The tables of contents of selected journals that are concerned mainly with EM or its subspecialties were reviewed back to 1995, or the publication start date of the journal, whichever was later, to identify articles that were relevant to the efficacy or value of the specialty of EM. In addition, relevant "landmark" articles already known to the authors that were published prior to 1995 or in other non-EM journals were included as well. Full text of these articles was then obtained through an electronic medical library system and the first author reviewed the text of each article to verify its relevance for inclusion. Articles were selected for inclusion if they showed a positive or beneficial effect of EM or of EM physician-provided medical care. Articles were then verified for inclusion by consensus of the authors, and all authors agreed on the inclusion of all the articles in the final compilation. Articles about prehospital care not provided by physicians were not included. The selected articles were grouped into nine different topic categories. Since this study was only comprised of a journal article review, it was exempt from institutional review board approval.

The journals reviewed included *Annals of Emergency Medicine*, *Academic Emergency Medicine*, the *American Journal of Emergency Medicine*, and the *Journal of Emergency Medicine *from August 2010 back to January 1995, *Prehospital and Disaster Medicine *from July 2010 back to January 2002, the *Western Journal of Emergency Medicine *and the *International Journal of Emergency Medicine *from July 2010 back to their start dates in 2008, and the *European Journal of Emergency Medicine *from October 2010 back to January 1995. Several other prominent EM journals were not reviewed for this study simply because the authors did not have electronic access to the full text of all their articles.

The decision to limit this review to primarily articles published after 1994 was based on consensus by the authors that many articles published prior to 1995 were either no longer directly relevant or the same subject content had been repeated in more recent publications.

Articles selected for inclusion in this review addressed a study or summary analysis related to showing the efficacy or value of EM or aspects of EM delivered care. Case reports and case series of successful clinical care in the Emergency Department (ED) were not included (there are of course thousands of these types of published reports in the medical literature). Articles were then subclassified into one of the nine categories listed below. If an article addressed more than one aspect of EM efficacy or effectiveness it was placed in the single category deemed most applicable by the reviewer. Each article was only placed into a single category even if it addressed more than one aspect of EM efficacy.

1. Efficacy of EM for critical care and procedures

2. Efficacy of EM for efficiency or cost of care

3. Efficacy of EM for public health and preventive medicine

4. Efficacy of EM for radiology (i.e., accuracy of reading films, etc.)

5. Efficacy of EM for trauma and airway management

6. Efficacy of EM for using ultrasound

7. Efficacy of EM faculty

8. Efficacy of EM residencies

9. Overviews and editorials of EM efficacy or value.

## Results

A total of 282 articles related to the efficacy of EM were identified with the following numbers of articles in each subcategory (see reference list for citation specifics):

1. Critical care and procedures: 31 [4-34]

2. Efficiency or cost of care: 30 [35-64]

3. Public health and preventive medicine: 34 [65-98]

4. Radiology: 11 [99-109]

5. Trauma and airway: 27 [110-136]

6. Ultrasound: 56 [137-192]

7. EM faculty: 34 [193-226]

8. EM residencies: 24 [227-250]

9. Overviews and editorials: 35 [251-285].

Of note, while not an aim of the study, the authors identified only three articles with negative evaluations of EM; these were not included in the final compiled list.

## Discussion

This review of selected medical literature since 1995 with the compilation of articles supporting the efficacy or value of EM shows that there are an extensive number of published references for each subcategory supporting the efficacy and value of EM. The content and conclusions of the articles in the sets identified above provide support for the following statements (listed in the same order as the topic categories above):

1. Trained emergency physicians can effectively and safely provide critical care and perform selected invasive procedures.

2. EM and care rendered in EDs offer many efficiencies and cost-effectiveness of care delivery within the broader healthcare system.

3. EM and EDs can provide a number of effective Public Health and Preventive Medicine measures.

4. Trained EM physicians can accurately and safely interpret radiographic studies.

5. Trained EM physicians can safely and effectively manage trauma patients and perform advanced airway management.

6. Trained EM physicians can safely and accurately perform and interpret ultrasound studies, both diagnostic and procedure-related.

7. EM faculty can deliver high-quality patient care and medical training, and are effective for patient safety.

8. Trained EM physicians can accurately interpret electrocardiograms.

9. EM residency training results in improved patient care in the ED.

10. EM is an important key component for all national healthcare systems.

The article contents and specific article conclusions found in this medical literature review and compilation provide literature-based support for the efficacy of EM and trained emergency physicians. We found that when EM is a distinct and recognized medical specialty with its own specialist training programs (residencies), there is supportive literature for the premise that EM contributes to effective, safe, efficient, and cost-effective patient care.

Limitations of this manuscript include the subjective method used for selection of articles, the subjective consideration of what "value" is, and that an electronic keyword article search was not performed. The authors had determined that as a practical matter this approach was required since using an electronic keyword search with the terms "Emergency Medicine" plus "Efficiency" or "Effectiveness" to identify relevant articles would have missed many of the articles that were identified, since many did not have the terms "efficacy" or "effectiveness" in their titles. A demonstration of the validity of this concern is the manuscript published in 2006 by Peter Hallas [286], which used a structured PubMed search and found only 25 articles on EM efficacy. His article's conclusions were similar to the ones listed above, and included "Having specialists in EM improved care for patients who need urgent treatment..." and "The establishment of a specialty in Emergency Medicine would most likely improve the standard of care for acutely ill patients."

Another limitation of the current manuscript is that only a limited number of journals were reviewed. So it certainly is likely that there are other relevant articles that demonstrate EM efficacy and effectiveness in other journals that were not reviewed. That such a large number of relevant articles were discovered in spite of this indicates that there may be many more articles in other or earlier journals that support the efficacy and effectiveness of EM, further strengthening the ten conclusion statements above. In addition, since each article was placed into only one category even though a number of the articles actually showed EM efficacy or effectiveness in more than one category, the number of supportive articles in most categories could be considered actually to be higher than the numbers shown in the Results section above.

Since the scientific quality or rigor of the articles included is variable, a strength of evidence analysis of all the included articles would be desirable as well, and should be the focus of future efforts, as should a review of the journals not included in this study. A follow-up goal of this project is to obtain permission from the journals from which the articles were selected to be able to disseminate full text versions of all of the articles as a resource maintained in conjunction with the International Federation for Emergency Medicine.

The authors want to emphasize that this study should be regarded as a preliminary and partial compilation of supportive literature in view of the above-noted study limitations. Also the authors readily acknowledge that, while they encountered only three negative studies compared to the hundreds of positive studies, the methodology was specifically designed to identify and collect articles that were positive toward EM, making the conclusions somewhat preordained.

## Conclusions

There is extensive medical literature support for the efficacy, effectiveness, or value for both EM as a medical specialty and for emergency patient care delivered by trained EM physicians.

## Competing interests

The authors declare that they have no competing interests.
